# MiR-30a: A Novel Biomarker and Potential Therapeutic Target for Cancer

**DOI:** 10.1155/2018/5167829

**Published:** 2018-08-06

**Authors:** Lin-hong Jiang, He-da Zhang, Jin-hai Tang

**Affiliations:** ^1^Department of Oncology, Xuzhou Medical University, Xuzhou, Jiangsu, China; ^2^Xuzhou Infectious Disease Hospital, Xuzhou, Jiangsu, China; ^3^Department of General Surgery, School of Medicine, Southeast University, Nanjing, Jiangsu, China; ^4^Department of General Surgery, The First Affiliated Hospital with Nanjing Medical University, Nanjing, Jiangsu, China

## Abstract

MicroRNAs (miRNAs) are small, highly conserved noncoding RNAs molecules, consisting of 18–25 nucleotides that regulate gene expression by binding to complementary binding sites within the 3′untranslated region (3′UTR) of target mRNAs. MiRNAs have been involved in regulating gene expression and diverse physiological and pathological processes. Several studies have reported that miR-30a, situated on chromosome 6q.13, is produced by an intronic transcriptional unit. Moreover, miR-30a has demonstrated its role in biological processes, including inhibiting proliferation and metastasis in many tumors, autophagy in chronic myelogenous leukemia, and regulating TGF-b1-induced epithelial-mesenchymal transition. However, based on the pathogenetic relationship between miR-30a and cancer in tumorigenesis, we believe that miR-30a may serve as tumor promising biomarker. Moreover, it would offer a therapeutic target for the treatment of cancer.

## 1. Introduction

Based on the latest world cancer statistics, global cancer burden is constantly rising. It was reported that it rises to 14.1 million new cases and 8.2 million cancer deaths occurred in 2012. The most common diagnoses were those of the lung cancer (1.8 million, 13.0% of the total), breast cancer (1.7 million, 11.9%), and colorectal cancer (1.4 million, 9.7%) worldwide [1].

More and more evidences have confirmed that miRNAs participate in diverse biological processes, which have a significant correlation with cancers [2]. MiRNAs are a class of small, highly conserved, and noncoding RNAs that regulate gene expression at posttranscriptional level by binding to the 3′ untranslated region (UTR) of target mRNAs. MiRNAs are involved in tumor proliferation, apoptosis, differentiation, invasion, and metastasis, as well as tumorigenesis [3, 4]. MiRNAs dysregulation is a general feature in many cancers [5], such as glioma [6] and papillary thyroid carcinoma [7],; miR-30a expression is overexpressed but downregulated in lung cancer [8, 9], giant cell tumor [10], breast cancer[11–15], renal cell carcinoma[16], hepatocellular carcinoma[17, 18], colorectal cancer[19, 20], ovary cancer[21], chondrosarcoma[22], gastric cancer[23, 24], urothelial carcinoma of the bladder[25], nasopharyngeal carcinoma[26], Ewing tumor[27], pancreatic cancer[28], prostate cancer[29], and cervical cancer[30]. This data implies that the miR-30 family may play different roles as oncogenes or tumor suppressor genes depending on the type of cancer; similarly, miR-30a also plays an important role in cancer development and progression by modulating target genes, including inhibiting proliferation, invasion, and migration, inducing apoptosis. Furthermore, chemotherapy is still an important treatment method for cancer, but the intrinsic or acquired drug resistance, especially multidrug resistance (MDR), is considered to be the major reason of the chemotherapy failure. However, it strongly stated an evidence that miR-30a increases the cisplatin sensitivity of gastric cancer cells by suppressing epithelial-to-mesenchymal transition (EMT) [31]. Therefore, miR-30a may be a potential therapeutic target for cancer treatment.

## 2. MiR-30a in Cell Proliferation

**Figure 1:**
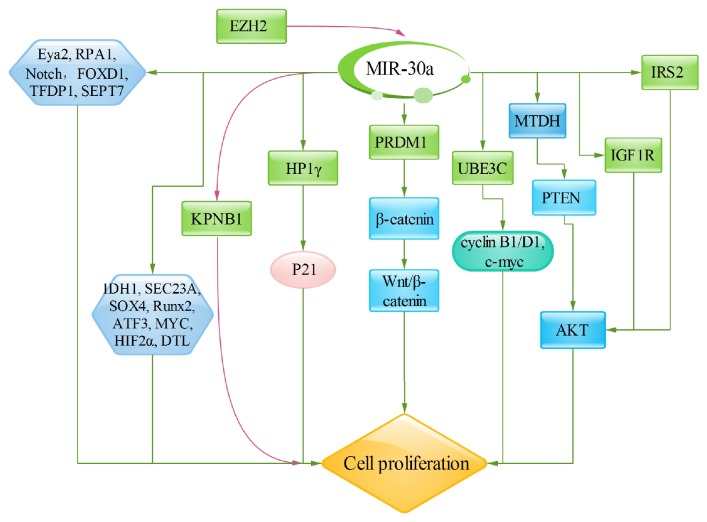
MiR-30a significantly inhibits cell proliferation. Several important signaling pathways are involved in cell proliferation, including Wnt/*β*-catenin, MTDH/PTEN/Akt, and PI3K signaling pathway. Similarly, miR-30a also regulates directly the target genes, such as Eya2, HP1*γ* (CBX3), EZH2, IGF1R, RPA1, Notch, IRS2, SOX4, Runx2, DTL, ATF3, MYC, HIF2*α*, and SEPT7, which are all involved in regulating cell proliferation procedure.

Several reports have confirmed that the miR-30a can significantly inhibit cancer cell proliferation [15, 18]. *β*-catenin is the key mediator of the canonical Wnt pathway; aberrant activation could lead to the accumulation and nuclear translocation of cytosolic *β*-catenin, that is, the symbol of the kinetic Wnt pathway [32, 33]. MiR-30a decreases cell proliferation by targeting PRDM1 served as downstream of a sequential RAWnt-Fgf signaling cascade, inhibiting the *β*-catenin expression in both the cytoplasm and nucleus [34]. Besides, the ubiquitin protein ligase E3C (UBE3C), a direct target of miR-30a-5p, plays important roles in cancers through the mutation in the HECT domain and Wnt/*β*-catenin signal pathway [35, 36]. MiR-30a-5p overexpression inhibits breast cancer cell proliferation by downregulating UBE3C, as well as cyclin B1, cyclin D1, and c-myc [14]. It is also found that miR-30a inhibits liver cancer cell proliferation by targeting MTDH/PTEN/Akt pathway [18]. Metadherin (MTDH) is situated on chromosome 8q22.5, which is a single-pass transmembrane protein consisting of 582 amino acids. MiR-30a overexpression significantly inhibits MTDH expression, which leads to increasing PTEN expression, and then inhibits Akt phosphorylation, concomitant with the inhibition of cell proliferation [18]. Other miR-30 mediated targets directly related to cell proliferation are Eya2, HP1*γ* (CBX3), EZH2, IGF1R, RPA1, Notch, and IRS2. The Eya proteins (Eya1–4) are the eyes absent gene product in Drosophila. Eyes absent is one member of the retinal determination network, which is involved in gonadogenesis, myogenesis, limb formation, neurogenesis, cell cycle control, thymus, and kidney development [11]. MiR-30a decreases cell proliferation by inhibiting Eya2 expression. HP1*γ* is one member of the mammalian heterochromatin protein 1 (HP1) family that contains HP1*α* (CBX5), HP1*β* (CBX1), and HP1*γ* (CBX3). HP1*γ* is situated to both heterochromatic and euchromatic regions [37]. It is found that HP1*γ*, posttranscriptionally targeted by miR-30a, promotes colorectal cancer cell proliferation by directly targeting p21 [19]. Enhancer of zeste homolog 2 (EZH2) is a histone-lysine N-methyltransferase and a polycomb group protein, and it is an important component of polycomb repressive complex 2 (PRC2), containing EED, SUZ12, and RbAP46/48 [38]. EZH2 knockdown upregulates miR-30a expression that directly increased miR-30a target KPNB1, decreasing malignant peripheral nerve sheath tumor cell proliferation [38]. IGF-IR is an important transmembrane receptor tyrosine kinase (RTK) on cell membrane surface, which is mainly activated by IGF1 or IGF2. The activated IGF1R combines to adaptor molecules and then stimulates downstream 3-kinase (PI3K)/Akt signaling pathway which mediates oncogenic transformation, growth, and survival of cancer cells [39, 40]. RPA plays a role in the replication of DNA, and it has been found that miR-30a hinders the replication of DNA and induces DNA fragmentation by targeting RPA1 and then slows cancer cell proliferation [21]. NOTCH signaling pathway is involved in cell proliferation procedure; miR-30a significantly inhibits cell proliferation by targeting Notch1 to inhibit the activity of Notch1. IRS2 (insulin receptor substrate 2), situated in the 13q34 region, is frequently overexpression in CRC; miR-30a inhibits the IRS2 expression and then diminishes the expression of Akt/p-Akt that mediates cell proliferation [41]. Moreover, it is also demonstrated that the antiapoptosis gene AVEN and the transcription factor-related genes FOXD1, TFDP1, IDH1, SEC23A, SOX4 [42], Runx2 [22], DTL, ATF3, MYC [43], HIF2*α* [44], and SEPT7 [6] are all involved in nonattachment growth via miR-30a [13] (Figure 1).

## 3. MiR-30a in Cell Apoptosis

Cells would be induced to apoptosis if the intracellular environment is persistent disorders or extremely in danger. Then miR-30a promotes cancer cell apoptosis by regulating several relevant effectors.

**Figure 2:**
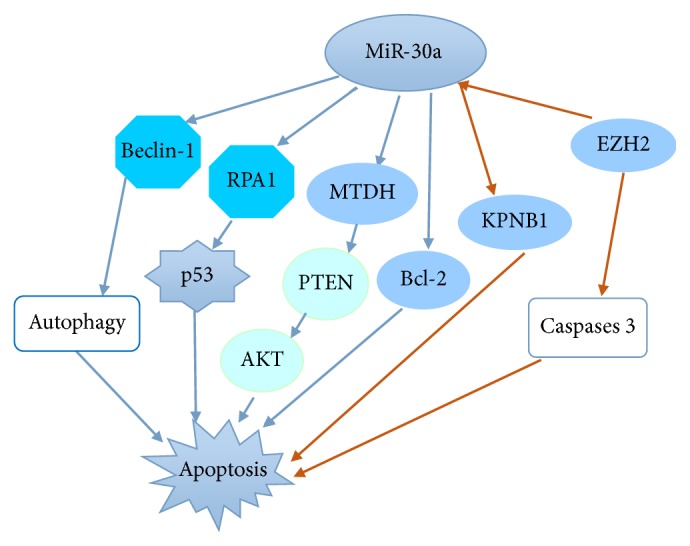
MiR-30a significantly promotes cell apoptosis by regulating several relevant effectors. MiR-30a is also involved in the controlling processes by Bcl-2, autophagy, increasing p53 expression, and inhibiting DNA replication. In addition, miR-30a induces cell apoptosis by MTDH/PTEN/AKT pathway.

Autophagy plays the vital physiological and pathological processes in sustaining cellular homeostasis; its activation helps cells clear up damaged proteins or organelles through lysosomal degradation, affording energy and nutrients for cells survival. Recent study has showed that miR-30a inhibits Beclin-1 activity, thereby hindering autophagic vesicle nucleation and autophagy initiation, thus promoting cell apoptosis or death [45]. Apoptosis is a process of multiple genes strictly controlled. These genes are highly conserved between species, such as Bcl-2 family and caspase family [46]. Overexpression of miR-30a downregulates the expression of BCL-2, enhancing cells apoptosis [6]. Additionally, downexpression of EZH2 upregulates miR-30a expression and miR-30a overexpression directly inhibits its target KPNB1, inducing cell apoptosis. Furthermore, downexpression of EZH2 increases cleaved caspase-3 signals. Therefore, it suggests that miR-30a promotes malignant peripheral nerve sheath tumor cell apoptosis by EZH2/miR-30a/KPNB1 signaling pathway [38]. Besides, miR-30a overexpression significantly inhibits MTDH protein expression, which increases PTEN expression, and then inhibits Akt phosphorylation, concomitant with inducing cell apoptosis [18]. In addition, miR-30a decreases RPA1 expression and then inhibits DNA replication, increasing p53 expression and inducing cell apoptosis [21] (Figure 2).

## 4. MiR-30a in Cell Cycle State

It is found that apoptosis accompanied with cell cycle arrest induced by miR-30a contributes to inhibiting cell proliferation. Similarly, it is also found that miR-30a inhibits cell cycle progression at the G0/G1 and G1/S transition.

**Figure 3:**
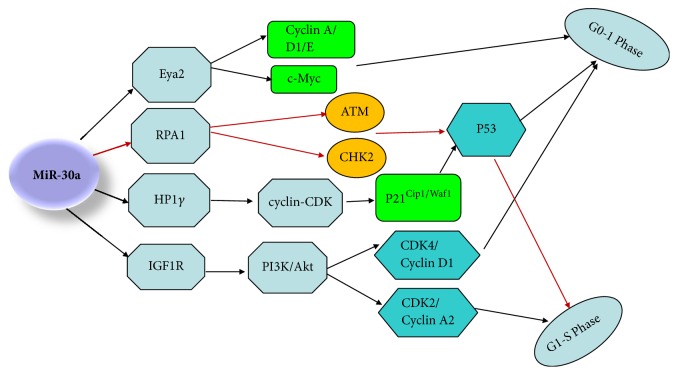
MiR-30a induces cell cycle arrest at the G0/G1 and G1/S in cancer. MiR-30a suppresses cell cycle progression almost by cyclin-CDK inhibitors and IGF1R-mediated PI3K/Akt pathway. MiR-30a induced p53 and p21^Cip1/Waf1^ protein expression to lead to DNA repair or DNA damage caused apoptosis. C-Myc, cyclin A, cyclin D1, and cyclin E participate in cell cycle arrest in G1 phase.

P21^Cip1/Waf1^ is a very important cyclin-CDK inhibitor that negatively mediates G1 phase progression [60]; some researches have reported that miR-30a specifically targets HP1*γ*, which inhibits colon cancer growth by upregulating P21^Cip1/Waf1^ expression, resulting in cell cycle arrest at G0/G1 in colorectal cancer cells. Similarly, several studies have also found that p53 expression is upregulated by knocking down HP1*γ* [19]. Besides, miR-30a is not only reported to cause DNA damage by blocking the IGF1R-mediated PI3K/Akt pathway but also can decrease DNA replication by targeting RPA1 in ovarian cancer and gastric cancer, and downexpression of RPA1 increases the phosphorylation of ATM and CHK2, which induces p53 expression and arrests the cells at G1/S-phase [21]. It is well known that p53 serves as a contributor to induce DNA repair or DNA damage caused apoptosis by transcriptional upregulation of the cyclin dependent kinase (CDK) active inhibitor p21^Cip1/Waf1^ [61]. More important, it is also found that miR-30a could regulate cell cycle through targeting IGF1R in nonsmall cell lung cancer, and IGF1R overexpression can upregulate expression of CDK4/Cyclin D1 and CDK2/Cyclin A2 complex via Akt signaling pathway, and IGF1R silencing hinders S/G2 transition by inhibiting the expression of CDK2/Cyclin A2 complex through Akt signaling pathway. Inhibiting of Akt signaling pathway could also hinder G1/S transition by inhibiting the expression of CDK4/Cyclin D1 complex. Finally, miR-30a also mediates G1 cell cycle arrest by targeting Eya2, concomitant with decreased c-Myc, cyclin A, cyclin D1, and cyclin E expression in breast cancer [11] (Figure 3).

## 5. MiR-30a in Invasion and Metastasis

**Figure 4:**
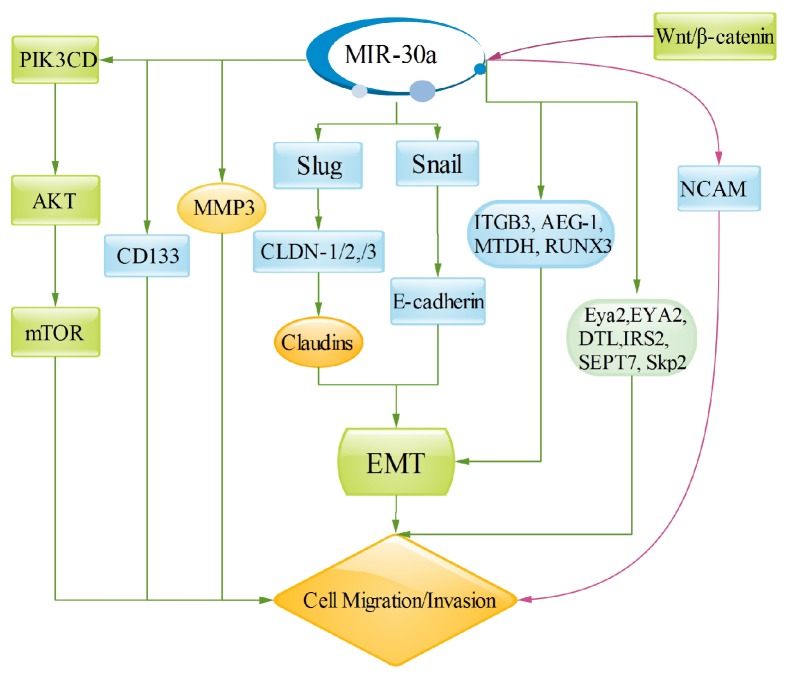
MiR-30a regulates target genes involved in cancer invasion and metastasis. Wnt signaling and PI3K/Akt pathways are major pathways to regulate cell invasion and metastasis. Partial target genes of miR-30a mediate EMT to control cell invasion and metastasis. In addition, Eya2, EYA2, DTL, IRS2, SEPT7, and Skp2 also mediate cancer cell invasion and migration. However, miR-30a promotes migratory and invasive abilities in glioma and pancreatic cancer cells.

Migration and invasion are critical processes for cells. MiR-30a has already been involved in regulating metastatic and invasive activity in various types of cancer. Wnt signaling pathway is frequently dysregulated in various tumor types and plays important roles in tumor development and progression, including regulating cell proliferation, invasion, and migration [16]. Wnt signaling consists of canonical and noncanonical arms, depending on the ligand; canonical Wnt signaling pathway (Wnt/*β*-catenin pathway) is initially executed by the bipartite transcription factor complex *β*-catenin/TCF (T cell factor) or LEF1 (lymphoid enhancer factor) to activate or inhibit the target genes, including c-Myc and cyclin D1 [16]. MiR-30a-5p is a novel downstream miRNA of Wnt/*β*-catenin pathway, which is activated by combining *β*-catenin/TCF4 with two sites in the promoter region of miR-30a-5p. Furthermore, miR-30a-5p increases cell invasion by directly targeting NCAM in glioma cells. Therefore, it suggests that the Wnt/*β*-catenin-miR-30a-5p-NCAM axis acts a vital role in glioma cells invasion [16]. PIK3CD, a target gene of miR-30a, is an important component of the PI3K/Akt pathway, and a study has shown that miR-30a overexpression downregulates the expression of Akt and mTOR and both of them phosphorylated forms; however, upregulation of PIK3CD rescues this effect. Thus, these results confirm that miR-30a inhibits cell migration and invasion by the inhibition of PI3K/Akt/mTOR signaling pathway [20]. Furthermore, miR-30a has an important correlation with EMT. The epithelial-to-mesenchymal transition (EMT) could lose tumor cells epithelial features transiently, including the loss of apicobasal polarity, disintegrating tight and adherent junctions, and obtaining mesenchymal traits, which leads to cell invasion and metastasis [47]. MiR-30a could promote claudins expression by increasing tight junction molecules, CLDN-1, CLDN-2, and CLDN-3 via targeting Slug, and then regulates EMT to control invasion and metastasis. And Snail is targeted by miR-30a, one inducer of EMT, which is considered the transcriptional suppressor of E-cadherin, which is also involved in cell invasion and migration [47, 49]. Besides, previous researches coupled *β*3 integrin to epithelial-mesenchymal transition (EMT) and metastasis, and miR-30a downregulates *β*3 integrin expression, which can inhibit the rewiring of Erk/Ets-1 signaling pathways, thereby inhibiting cell migration and invasion [48]. Similarly, ITGB3 [51], Astrocyte Elevated Gene-1 (AEG-1) [8], metadherin (MTDH) [15], and RUNX3 [23], they are all connected with EMT that regulates cell invasion and migration through targeted miR-30a. MMPs are family members of extracellular proteinases that regulate cellular biological processes, including cell proliferation, invasion, and migration [62, 63]. It is reported that miR-30a could reduce hepatocellular carcinoma cell invasion and migration by downregulating MMP3 expression [17]. Consequently we can conclude that miR-30a could regulate biomarker of EMT and MMPs to inhibit cell invasion and migration, including downregulating the protein levels of vimentin and MMP3 and upregulating the E-cadherin protein level. Furthermore, miR-30a also inhibits breast cancer cell migration through inhibiting Eya2 [11], EYA2 [52], DTL [53], insulin receptor substrate 2 (IRS2) [42], SEPT7 [6], and Skp2 [54] expression. However, it is also found that miR-30 family is modulated by CD133 and promotes migratory and invasive abilities in pancreatic cancer cells, which is opposite of miR-30a function (Figure 4).

## 6. MiR-30a Regulates Drug Susceptibility through EMT Signaling

**Figure 5:**
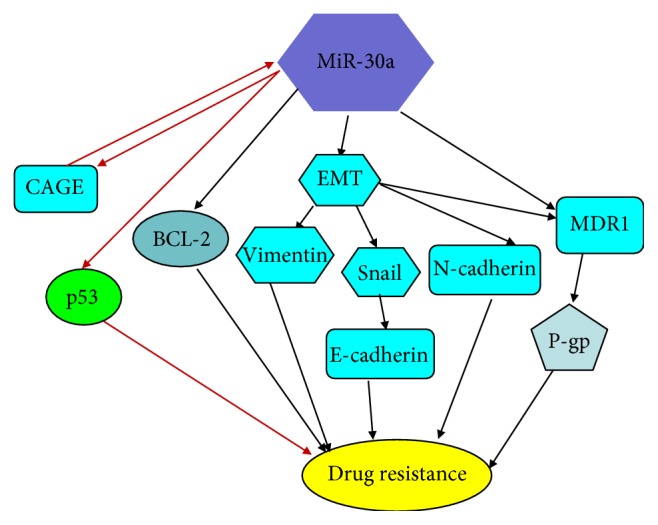
MiR-30a regulates drug susceptibility through EMT signaling, P-gp signaling, and other relevant target genes. EMT not only regulates cell invasion and metastasis but also participates in drug resistance. In cisplatin-resistant gastric cancer cells, the expressions of Snail and Vimentin are higher, but with lower expression of miR-30a and E-cadherin expression. However, the loss of epithelial markers and mesenchymal markers is correlated with MDR development. Besides, miR-30a overexpression increases paclitaxel sensitivity by inhibiting BCL-2 expression in nonsmall cell lung cancer. In addition, CAGE expression promotes miR-30a expression, which inhibits p53 expression, increasing drug resistance by forming miR-30a-CAGE-p53 feedback loop.

EMT not only acts an important role in regulating cell invasion and metastasis [47, 64, 65] but also participates in drug resistance, for example, miR-203 could enhance drug susceptibility through inhibiting EMT via targeting Snail2 [66]. Snail and Slug/Snail2 are important EMT-inducing transcription factors, which could directly bond and inhibit E-cadherin transcription, which mediates drug resistance [64]. Recently it is found that the expression of miR-30a in cisplatin-sensitive of gastric cancer cells is higher than that in cisplatin-resistant cells; and compared with cisplatin-sensitive of gastric cancer cells, the expressions of Snail, Slug, and Vimentin are higher, but with lower expression of E-cadherin in the cisplatin-resistant cells, which implies that EMT is associated with drug resistance in gastric cancer cells [31, 67]. Subsequently, overexpression of miR-30a inhibits the expression of Snail and Vimentin but promotes E-cadherin expression [31], which implies that miR-30a could enhance cisplatin sensitivity by inhibiting EMT in gastric cancer cells. Multidrug resistance (MDR) is considered to be the major reason of the chemotherapy failure, and several studies have confirmed that the loss of epithelial markers and mesenchymal markers is correlated with MDR development [31, 68, 69]. P-gp is a transmembrane glycoprotein, encoded by MDR1 gene [70]. Furthermore, overexpression of miR-30a increases E-cadherin but decreases the expression of N-cadherin and P-gp in the cisplatin-resistant cells [67], which may suggest that miR-30a could enhance cisplatin sensitivity by regulating MDR via mediating EMT in the cisplatin-resistant gastric cancer cells (Figure 5).

## 7. MiR-30a Regulates Drug Susceptibility through Inhibiting BCL-2 and p53

In addition, BCL-2, the target gene of miR-30a, an important apoptosis regulator, contributes to paclitaxel resistance in nonsmall cell lung cancer [71–73]. It is found that miR-30a overexpression increases paclitaxel sensitivity by decreasing BCL-2 expression, promoting cell apoptosis; similarly, BCL-2 overexpression increases paclitaxel resistance, reducing miR-30a expression. This data implies that miR-30a regulates paclitaxel susceptibility via inhibiting BCL-2 [73]. CAGE is a cancer antigen and shows resistance to microtubule-targeting drugs though mediating p53 expression [74]. In addition, it is found that CAGE has a higher expression in celastrol resistance melanoma cell than celastrol sensitivity cells, but lower expression of p53 [55]. Similarly, it is also found that miR-30a increases CAGE expression, concomitant with inhibiting p53 expression in Malme3M cells [55]. And it is also confirmed CAGE and p53 combine with the promoter sequences of miR-30a by ChIP assays; in addition, p53 is confirmed to be a target of miR-30a by luciferase activity assays [55]. This data implies that CAGE promotes miR-30a expression by combining to the promoter sequences of miR-30a, which inhibits p53 expression that increases drug resistance and decreases cell apoptosis by forming miR-30a-CAGE-p53 feedback loop (Figure 5).

## 8. MiR-30a in PIN

Many miRNAs have been confirmed to be related to the progression and carcinogenesis in cancer. However, the latest researches show that miRNA-regulated protein interaction networks (PINs) were built by confident pairs and known interaction data in the human protein reference database (HPRD) [75]. The latest data shows that let-7c has been constructed a more detailed let-7c-regulated PINs to act as breast cancer diagnostic markers [76, 77]. Based on the known biological processes of miR-30a, we have constructed the mutual correlation among of proliferation, apoptosis, invasion, and metastasis through related target genes (Table 1). By miRNA-regulated PINs, we can more explicitly show the identification of cancer diagnostic markers.

## 9. Conclusion

In this review, it is learned that miR-30a acts as a tumor suppressor to regulate various biological processes, including proliferation, invasion, metastasis, and apoptosis. Most important of all, miR-30 is also involved in several signal pathways or target genes to regulate drug susceptibility. Thus, it is advised that miR-30a could be regarded as a promising biomarker and potential therapeutic target in cancer development and progression and may offer a new insight into the treatment of cancer, although there is still a long way to apply into clinic.

## Figures and Tables

**Table 1:** Target genes of miR-30a in response to cancer cells.

Target gene	Cancer type	References
SEPT7	Glioma cancer	Jia Z et al. [6]
AEG-1	lung cancer	Liu K et al. [8]
Eya2	Breast cancer	Fu J et al. [11]
Vimentin	Breast cancer	Cheng CW et al. [12]
UBE3C	Breast cancer	Xiong J et al. [14]
NCAM	glioma	Wang Z et al. [16]
MTDH	liver cancer	Li WF et al. [18]
HP1*γ*	Colorectal Cancer	Liu M et al. [19]
PIK3CD	Colorectal Carcinoma	Zhong M et al. [20]
RPA1	ovarian cancer, gastric cancer	Zou Z et al. [21]
	
Runx2	chondrosarcoma	Jiang D et al. [22]
RUNX3	gastric cancer	Liu Z et al [23]
Notch1	urothelial carcinoma	Zhang C et al. [25]
	of the bladder	
E-cadherin	nasopharyngeal carcinoma	Wang HY et al. [26]
EWS-FLI1	Ewing tumor	Franzetti GA et al. [27]
CD99	Ewing tumor	Franzetti GA et al. [27]
PRDM1.	glioma	Wang X et al. [34]
EZH2	malignant peripheral	Zhang P et al. [38]
	nerve sheath tumor	
IGF1R	in non-small cell lung cancer,	Wen XP et al. [40],
IRS2	Colorectal cancer	Zhang Q et al. [41]
SOX4	chondrosarcoma	Lu N at al. [42]
Slug	Breast cancer	Chang CW et al. [47]
*β*3 integrin	Breast cancer	Li W et al. [48]
Snai1	lung cancer,	Kumarswamy R et al. [49],
	hepatocellular carcinoma	Liu Z et al. [50]
ITGB3	Colorectal Carcinoma	Wei W et al [51]
EYA2	lung cancer	Yuan Y et al. [52]
DTL	colon carcinoma	Baraniskin A et al. [53]
Skp2	Pulmonary Vascular	Qi F et al. [54]
	Hyperpermeability	
p53	hepatocellular carcinoma	Park D et al. [55]
HNF4*γ*	intestinal metaplasia	Sousa JF et al. [56]
ET_A_R	ovarian carcinoma	Sestito R et al. [57]
beclin 1	lung cancer, Breast cancer	Zhu H et al. [58]
FOXL2	ovarian	Wang T, et al. [59]
